# ‘Extended’ restricted kinematic alignment results in decreased residual medial gap tightness among osteoarthritic varus knees during robotic-assisted total knee arthroplasty

**DOI:** 10.1302/2633-1462.58.BJO-2024-0054.R1

**Published:** 2024-08-02

**Authors:** Krishna K. Eachempati, Apurve Parameswaran, Vinay K. Ponnala, Apsingi Sunil, Neil P. Sheth

**Affiliations:** 1 Department of Orthopaedics, Medicover Hospitals, Hyderabad, India; 2 Penn Orthopaedics at Pennsylvania Hospital, University of Pennsylvania, Philadelphia, Pennsylvania, USA

**Keywords:** Restricted kinematic alignment, Extended restricted kinematic alignment, Robot-assisted total knee arthroplasty, Total knee arthroplasty, Image free robot, Gap balancing, robotic-assisted total knee arthroplasty, osteoarthritic varus knees, knees, Soft-tissue releases, total knee arthroplasty (TKA), flexion, soft-tissue balancing, varus osteoarthritis, hip-knee-ankle angle, tibial component

## Abstract

**Aims:**

The aims of this study were: 1) to describe extended restricted kinematic alignment (E-rKA), a novel alignment strategy during robotic-assisted total knee arthroplasty (RA-TKA); 2) to compare residual medial compartment tightness following virtual surgical planning during RA-TKA using mechanical alignment (MA) and E-rKA, in the same set of osteoarthritic varus knees; 3) to assess the requirement of soft-tissue releases during RA-TKA using E-rKA; and 4) to compare the accuracy of surgical plan execution between knees managed with adjustments in component positioning alone, and those which require additional soft-tissue releases.

**Methods:**

Patients who underwent RA-TKA between January and December 2022 for primary varus osteoarthritis were included. Safe boundaries for E-rKA were defined. Residual medial compartment tightness was compared following virtual surgical planning using E-rKA and MA, in the same set of knees. Soft-tissue releases were documented. Errors in postoperative alignment in relation to planned alignment were compared between patients who did (group A) and did not (group B) require soft-tissue releases.

**Results:**

The use of E-rKA helped restore all knees within the predefined boundaries, with appropriate soft-tissue balancing. E-rKA compared with MA resulted in reduced residual medial tightness following surgical planning, in full extension (2.71 mm (SD 1.66) vs 5.16 mm (SD 3.10), respectively; p < 0.001), and 90° of flexion (2.52 mm (SD 1.63) vs 6.27 mm (SD 3.11), respectively; p < 0.001). Among the study population, 156 patients (78%) were managed with minor adjustments in component positioning alone, while 44 (22%) required additional soft-tissue releases. The mean errors in postoperative alignment were 0.53 mm and 0.26 mm among patients in group A and group B, respectively (p = 0.328).

**Conclusion:**

E-rKA is an effective and reproducible alignment strategy during RA-TKA, permitting a large proportion of patients to be managed without soft-tissue releases. The execution of minor alterations in component positioning within predefined multiplanar boundaries is a better starting point for gap management than soft-tissue releases.

Cite this article: *Bone Jt Open* 2024;5(8):628–636.

## Introduction

Multiple alignment strategies for total knee arthroplasty (TKA) have been described.^1^ Mechanical alignment (MA) targets neutral distal femoral and proximal tibial resection, resulting in an unconstitutional state in most patients, and frequent gap imbalances requiring soft-tissue releases.^2-5^ Kinematic alignment (KA) aims to restore constitutional alignment using anatomical rather than mechanical bone resection, and rarely results in gap imbalances requiring soft-tissue releases.^6-9^ Restricted kinematic alignment (rKA) sets safe boundaries for KA to avoid recreating suboptimal biomechanics, while permitting minor adjustments in component positioning in the coronal plane, consistent with the native knee.^2^ KA and rKA focus on retention of native femoral anatomy, and restoration of physiological lateral compartment laxity.^2^

With the advent of robotic-assisted systems, calibration of bony resections with real-time quantification of their effect on the gap status has been rendered possible.^10,11^ Robotic-assisted TKA (RA-TKA) enables precise adjustment of component positioning in multiple planes, resulting in increased attention towards alternative alignment strategies. Moreover, RA-TKA offers the potential for precisely implementing established alignment options including MA, KA, and rKA.^10^ Additionally, the use of robotic technology expands the scope of conventional rKA through the use of supplemental boundaries in the sagittal and transverse planes, thereby preventing re-creation of outlier anatomy, while achieving enhanced gap balancing and retaining physiological laxity. This led to the development of a novel alignment strategy by the authors: ‘extended rKA’ (E-rKA).

We hypothesized that the use of E-rKA during RA-TKA would result in improved gap balance when compared with MA in the same set of knees. Consequently, a large proportion of knees could be managed without soft-tissue releases beyond routine surgical exposure. We also hypothesized that minor adjustments in component positioning compared with soft-tissue releases would be a better starting point for achieving gap balance during RA-TKA. The aims of this study were: 1) to describe the technique of E-rKA during RA-TKA; 2) to compare residual medial compartment tightness following virtual surgical planning using MA and E-rKA, in the same set of osteoarthritic varus knees; 3) to assess the requirement of soft-tissue releases during RA-TKA using E-rKA; and 4) to compare the accuracy of surgical plan execution between knees managed with adjustments in component positioning alone, and those which require additional soft-tissue releases.

## Methods

### Patient selection and preoperative evaluation

Following approval from the Institutional Ethics Committee from Medicover Hospitals, Hyderabad, India, an observational study was conducted. Informed consent was taken from all study participants. A total of 200 patients who underwent RA-TKA between January and December 2022 for primary osteoarthritis with an intra-articular varus deformity were recruited. Demographic data of all patients were collected. Preoperative weightbearing anteroposterior long-leg and full-leg (including knee) lateral radiographs were obtained. The mechanical lateral distal femoral angle (mLDFA), mechanical medial proximal tibial angle (mMPTA), limb alignment defined by the hip-knee-ankle angle (HKAA), and posterior tibial slope (PTS) were assessed using previously described techniques.^12-14^ Knee phenotype was assessed using the CPAK (coronal plane alignment of knee) classification.^15^ All surgeries were performed by a single surgeon (KKE) at a high-volume tertiary care hospital (Medicover Hospitals), using the NAVIO 7.0 (Smith & Nephew, UK) image-free robot. All patients received Legion implants (Smith & Nephew). Cruciate-retaining components were used wherever possible, while cruciate-substituting components were used where the posterior cruciate ligament (PCL) had to be sacrificed.

### Surgical exposure, and planning of MA

The mid-vastus approach was used for surgical exposure. The anterior capsule and deep fibres of the medial collateral ligament were released. Osteophytes and loose bodies were removed. Bony landmarks and the centres of the hip, knee, and ankle were registered on the robotic system. 3D mapping of the distal femur and proximal tibia was performed, creating a free-collection mesh. The angle between the anatomical transepicondylar axis (aTEA) and the posterior condylar axis (PCA) was assessed on the mesh. The PCA was then used as the reference for setting the desired femoral component rotation (FCR).

**Fig. 1:**
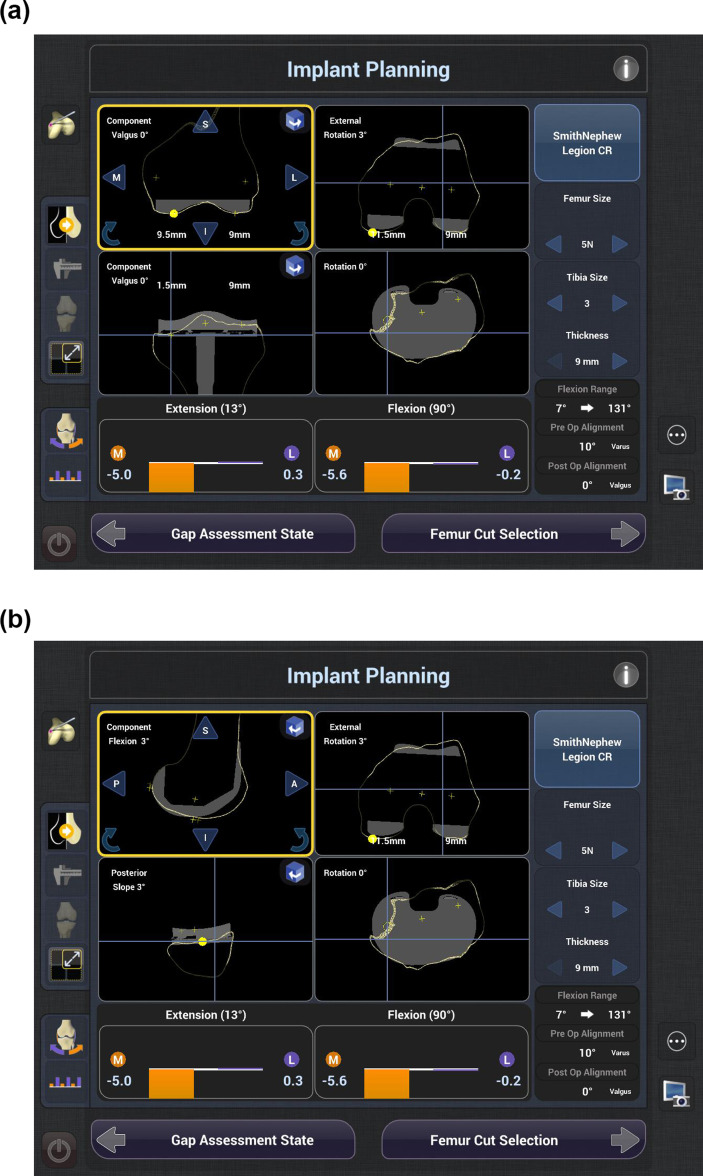
a) The surgical plan based on mechanical alignment, with the coronal and axial views of the planned femoral and tibial component positioning. b) The surgical plan based on mechanical alignment, with the sagittal and axial views of the planned femoral and tibial component positioning.

The robot planned the distal femoral and proximal tibial resections perpendicular to their respective mechanical axes, at a level based on the manufacturer’s recommendation for the specific implant. The smallest femoral component size which would restore posterior condylar offset without causing anterior notching, and the tibial component size which most closely matched the mediolateral dimension of the mesh, were planned. The default values for femoral component flexion (FCF), PTS, and FCR from the PCA were 3° each. Medial and lateral gaps throughout varus- and valgus-stressed range of motion were assessed. The robot presented the ‘default plan’ and the residual gap status in full extension and 90° of flexion (Figure 1).

The surgeon balanced the lateral compartment, with the aim of creating approximately 0 mm laxity in full extension and 90° of flexion. This was accomplished by altering the distal femoral resection level or femoral component size for isolated extension or flexion gap imbalance respectively, and/or the proximal tibial resection level or the polyethylene insert size for gap imbalance in both extension and flexion. The residual medial compartment tightness remaining to be corrected was noted.

### Planning and execution of the surgery using E-rKA

The robot permitted 28 types of alterations in the ‘default’ surgical plan, along 14 degrees of freedom (Table I). The boundaries in Table II were employed. The tibial base-plates used in the study had an asymmetric design, and were co-aligned with Akagi’s line, to ensure optimal coverage. The surgeon focused on retaining native femoral anatomy wherever possible, while performing minor alterations in component positioning within the predefined boundaries on the tibial side, or the femoral side in cases with outlier anatomy. The aim was to reduce medial tightness if it was greater than 4 mm, while maintaining lateral laxity of approximately 0 mm and 1 mm in extension and 90° of flexion, respectively.

**Table I.** The 28 types of alterations to the default surgical plan, in 14 degrees of freedom permitted by the robot.

Adjustment permitted in component positioning	Femoral component	Tibial component
Displacement along coronal plane	Medial displacement Lateral displacement	Medial displacement Lateral displacement
Displacement along sagittal plane	Superior displacement Inferior displacement	Superior displacement Inferior displacement
Displacement along transverse plane	Anterior displacement Posterior displacement	Anterior displacement Posterior displacement
Rotation in coronal plane	Varus rotation Valgus rotation	Varus rotation Valgus rotation
Rotation in sagittal plane	Flexion Extension	Flexion Extension
Rotation in transverse plane	Internal rotation External rotation	Internal rotation External rotation
Alteration of component size	Increase in size Decrease in size	Increase in size Decrease in size

**Table II.** The boundaries used for extended restricted kinematic alignment during robotic-assisted total knee arthroplasty.

Plane	Boundaries
Coronal	mLDFA : 86° to 93°, or 87° to 93°* mMPTA : 86° to 93°, or 87° to 93°* HKAA : 5° varus to 4° valgus, or 4° varus to 3° valgus*
Sagittal	FCF : 0° to 5° PTS : 2° to 7° FCF + PTS : ≤ 10°
Transverse	FCR : 1° external rotation to 5° internal rotation from the aTEA

* Narrower boundaries were used for patients who were aged above 80 years, or had a history of insufficiency fractures or medically treated osteoporosis, to lower the risk of implant subsidence.

FCF, femoral component flexion; FCR, femoral component rotation; HKAA, hip-knee-ankle angle; mLDFA, mechanical lateral distal femoral angle; mMPTA, mechanical medial proximal tibial angle; PTS, posterior tibial slope.

Based on the authors’ preliminary experience with the robot, prebalancing was not performed, as it usually resulted in the need for a thick polyethylene insert. The medial compartment was left up to 4 mm tight, while bony resections were planned to equalize medial gap tightness in extension and flexion. Following excision of the residual portions of the menisci and posterior osteophytes, most patients with a post-planning residual medial tightness up to 4 mm did not require soft-tissue releases for gap balancing. The lateral compartment laxity also increased to a more physiological value of up to 1 mm and 2 mm in extension and flexion, respectively.

**Fig. 2:**
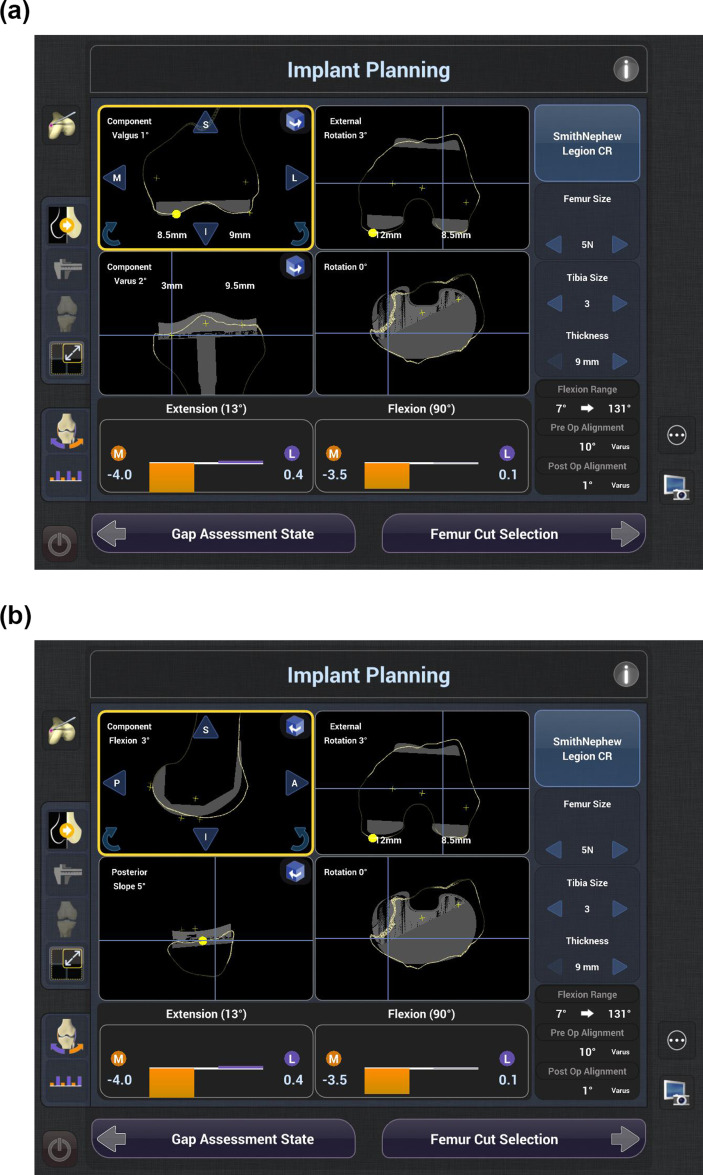
a) The final surgical plan based on extended restricted kinematic alignment (E-rKA), with the coronal and axial views of the planned femoral and tibial component positioning. b) The final surgical plan based on E-rKA, with the sagittal and axial views of the planned femoral and tibial component positioning.

Component size was adjusted where necessary, to match the shape and size of the mesh. Medial tightness in extension or flexion was addressed by altering the coronal angulation of the tibial component in a direction consistent with the native knee, and/or altering the PTS, respectively. Symmetrical tightness in extension and/or flexion was addressed by altering the proximal tibial resection level and/or the PTS, within the predefined boundaries. Bone resection was carried out using the ‘all-burr technique’. Postoperative gap status was reviewed with trial components in situ. Where required, soft-tissue releases were performed sequentially. The surgical plans were saved as screenshots in a password-protected robotic system database (Figure 2).

### Statistical analysis

Based on a pilot study at the institution, the SD of mean residual medial tightness during RA-TKA was approximately 3 mm. To detect a reduction in medial gap tightness of 1 mm, for a study power of 90% and significance at 0.05, a minimum of 189 samples was required. Statistical analysis was performed using SPSS software version 29 (IBM, USA). Means with SDs were calculated for numerical variables. Proportions of categorical variables were estimated. The Shapiro-Wilk test was used to assess normality of distribution. Levene’s test was used to assess homogeneity of variance. Means of numerical variables were compared using the paired or independent-samples *t*-test, as applicable. Pearson’s correlation coefficient was used to measure linear correlation between numerical variables.

Gap status, bone resection, mLDFA, mMPTA, FCF, PTS, FCR, and HKAA were compared using MA and E-rKA plans. Two subsets of patients who did (group A) or did not (group B) require soft-tissue releases beyond surgical exposure for attaining gap balance were identified, and compared for preoperative HKAA, planned HKAA, postoperative HKAA, planned axis correction ((preoperative HKAA) – (planned HKAA)), and error in postoperative alignment ((postoperative HKAA) – (planned HKAA)). The association between the planned axis correction and errors in postoperative alignment was assessed.

## Results

The study population comprised 142 (71%) females and 58 (29%) males; their mean age was 63.1 years (SD 7.91). Among them, 183 patients (91.5%) had an apex-distal knee phenotype, while 17 (8.5%) had an apex-neutral phenotype based on the CPAK classification.^15^ A significant reduction in medial gap tightness in extension and flexion was noted when E-rKA was planned in place of MA. A comparison of the gap status following virtual surgical planning using MA and E-rKA is summarized in Table III. The differences in planned bony resection in MA and E-rKA are shown in Table IV. E-rKA, when compared with MA, resulted in decreased posterior femoral resection in the medial compartment, but increased bone resection from the distal femur and proximal tibia medially, and the distal femur laterally.

**Table III.** A comparison of the gap status following virtual surgical planning during robotic-assisted total knee arthroplasty, using mechanical alignment and extended restricted kinematic alignment.

Compartment (position)	Planned gap status		
	Mean residual gap (MA), mm (SD; range)	Mean residual gap (E-rKA), mm (SD; range)	p-value
Medial (in extension)	-5.16 (3.10; 0.00 to -13.80)	-2.71 (1.66; -0.20 to -7.50)	< 0.001
Medial (in flexion)	-6.27 (3.11; 0.00 to -16.50)	-2.52 (1.63; -0.20 to -9.90)	< 0.001
Lateral (in extension)	0.30 (0.28; 0.00 to 1.00)	0.36 (0.26; 0.00 to 1.00)	< 0.001
Lateral (in flexion)	0.41 (0.27; 0.00 to 1.00)	0.79 (0.14; 0.60 to 1.20)	< 0.001

Negative values indicate tightness, while positive values indicate laxity.

* Paired *t*-test.

E-rKA, extended restricted kinematic alignment; MA, mechanical alignment.

**Table IV.** Differences in various planned bony resections during robotic-assisted total knee arthroplasty, using mechanical alignment and extended restricted kinematic alignment.

Resection	Mean (MA), mm (SD; range)	Mean (E-rKA), mm (SD; range)	p-value
Distal femur, medial	8.26 (1.33; 4.0 to 10.0)	8.89 (1.67; 5.0 to 14.0)	< 0.001
Distal femur, lateral	8.68 (1.21; 4.0 to 10.0)	9.54 (1.18; 6.0 to 12.0)	< 0.001
Proximal tibia, medial	2.06 (1.97; 0.0 to 7.5)	3.40 (1.86; 0.0 to 8.0)	< 0.001
Proximal tibia, lateral	8.92 (0.45; 8 to 10)	8.95 (0.89; 6.0 to 11.5)	0.663
Posterior femur, medial	11.59 (1.55; 8.0 to 15.0)	10.12 (0.61; 9.5 to 12.5)	< 0.001
Posterior femur, lateral	9.78 (0.93; 7.0 to 13.0)	9.57 (1.53; 6.0 to 15.0)	0.056

* Paired *t*-test.

E-rKA, extended restricted kinematic alignment; MA, mechanical alignment.

Significantly lower values of mLDFA, mMPTA, FCF, and FCR were noted while planning E-rKA, but a higher mean PTS was used (Table V). Overall, 156 patients (78%) were managed without soft-tissue releases beyond routine surgical exposure (Table VI). The required soft-tissue releases among the study participants are outlined in Table VI. Limb alignment was restored within the predefined boundaries in all patients. Table VII demonstrates the differences in alignment-related parameters between patients in groups A and B. No association between planned axis correction and errors in postoperative HKAA was noted (Table VII). There was no evidence of intraoperative femoral notching or patellar maltracking.

**Table V.** Preoperative and planned angular parameters using mechanical alignment and extended restricted kinematic alignment, during robotic-assisted total knee arthroplasty.

Angle	Mean preoperative value, ° (SD; range)	Mean planned value using MA, ° (SD; value)	Mean planned value using E-rKA, ° (SD; range)	p-value*
mLDFA	90.27 (2.19; 85 to 95)	90° (0°; fixed value of 90º)	89.43 (1.10; 86 to 93)	< 0.001
mMPTA	83.56 (3.29; 72 to 90)	90° (0°; fixed value of 90º)	87.61 (0.83; 86 to 90)	< 0.001
FCF	-	3° (0°; fixed value of 3º)	2.19 (1.44; 0 to 5)	< 0.001
PTS	11.27 (3.51; 3 to 16)	3° (0°; fixed value of 3º)	4.91 (1.07; 3 to 7)	< 0.001
FCR	-	3° (0°; fixed value of 3º)	0.62 (0.82; 0 to 3)	< 0.001
HKAA	-8.33 (3.28; -18 to -1)	0° (0°, fixed value of 0º)	-1.82 (1.23; -5 to -1)	< 0.001

Negative hip-knee-ankle angle values indicate varus, while positive values indicate valgus.

* Paired *t*-test.

FCF, femoral component flexion; FCR, femoral component rotation; HKAA, hip-knee-ankle angle; mLDFA, mechanical lateral distal femoral angle; mMPTA, mechanical medial proximal tibial angle; PTS, posterior tibial slope.

**Table VI.** Requirement of soft-tissue releases in the study population.

Soft-tissue release/additional procedures required	Patients, n (%)
None, except routine surgical exposure	156 (78)
Posteromedial corner with or without posterior capsular release	44 (22)
PCL recession	3 (1.5)
PCL release	2 (1.0)
Medial sliding femoral condylar osteotomy	1 (0.5)

PCL, posterior cruciate ligament.

**Table VII.** Overall and group-wise alignment related parameters during robotic-assisted total knee arthroplasty.

Parameter	Overall	Group A	Group B	p-value*
Mean preoperative HKAA, ° (SD; range)	-8.33 (3.28; -18 to -2)	-7.93 (3.00; -15 to -2)	-8.44 (3.35; -18 to -2)	0.363
Mean HKAA, planned, E-rKA, ° (SD; range) ↓ Mean postoperative HKAA, ° (SD; range)	-1.82 (0.92; -5 to -1) ↓ -2.14° (1.35; -5 to 0) (p = 0.001)‡	-1.80 (0.93; -5 to -1) ↓ -2.33 (1.50; -5 to 0) (p = 0.013)‡	-1.83 (0.92; -5 to -1) ↓ -2.09 (1.31; -5 to 0) (p = 0.009)‡	0.841 0.364
Mean planned axis correction, ° (SD; range)	6.51 (2.93; 0 to 15)	6.14 (2.84; 0 to 12)	6.62 (2.96; 0 to 15)	0.340
Mean error in postoperative alignment, ° (SD)	0.32 (1.22)	0.53 (1.25)	0.26 (1.22)	0.328
Association between planned axis correction and error in postoperative HKAA	*r* = 0.001† (p = 0.986)	*r* = 0.153† (p = 0.322)	*r* = -0.034† (p = 0.673)	-

Negative hip-knee-ankle angle values indicate varus, while positive values indicate valgus.

* independent-samples *t*-test.

† Pearson’s correlation coefficient.

‡ Paired *t*-test.

E-rKA, extended restricted kinematic alignment; HKAA, hip-knee-ankle angle.

## Discussion

E-rKA, as described by the authors, represents an evolution of the rKA concept, made possible through the use of robotic-assisted systems. It was found to be a practical and reproducible alignment strategy during RA-TKA, restoring all knees within the predefined boundaries. E-rKA follows the basic principles of rKA, but takes it one step further by setting additional boundaries in the sagittal and transverse planes. It uses KA as a starting point for component positioning and aims to restore physiological lateral laxity, unlike functional alignment, which uses MA as a starting point for surgery with the aim of creating rectangular gaps.^16^

The primary finding of this study was that E-rKA compared with MA resulted in reduced residual medial compartment tightness in both extension and flexion during RA-TKA in the same set of osteoarthritic varus knees (Table III). Similar to the findings of Vendittoli et al,^2^ most patients (78%) in the study population were managed using alterations in component positioning alone (Table VI). Soft-tissue releases during TKA unfavourably affect clinical outcomes.^17^ The elimination of the need for soft-tissue releases in 78% of the patients would improve their likelihood of achieving optimal clinical outcomes. Given the narrow boundaries of E-rKA, however, preparedness for the requirement of all types of soft-tissue releases would be appropriate. The improvement in gap balance by the use of E-rKA instead of MA is understandable, since a part of the onus of attaining balance is transferred from soft-tissue releases to component positioning. Our study demonstrates and quantifies this improvement in the same set of knees, using robotic technology, for the first time.

Another important finding of this study was that a significant difference was noted between planned and achieved alignment among patients in group A, as well as group B (Table VII). Patients in group A had a larger mean error in postoperative alignment. This increased error could be indicative of the unpredictability of the effect of soft-tissue releases on alignment. When compared between the two patient groups, however, the difference in errors did not reach statistical significance due to their high variance and the small number of patients in group A. Manual soft-tissue release is subjective and lacks precision.^18^ Over-correction or under-correction of deformities cannot be entirely prevented while performing soft-tissue releases, though accuracy may vary between surgeons. The authors believe that alterations in component positioning within predefined boundaries, rather than performance of soft-tissue releases, would be a better starting point for the correction of residual gap imbalance during TKA.

An association between planned axis correction and errors in postoperative alignment was sought, to explore a potential contributory role of large magnitudes of deformity correction, towards the observed errors. No association was found, however (Table VII). This would indicate that the execution of bony resection, as well as soft-tissue release, is associated with a certain constant error, which could be robot- or surgeon-specific, but is not dependent on the magnitude of deformity correction required. The robotic system is associated with an additional potential error up to 0.5° since it uses rounded off integers to quantify alignment. The sum total of these errors in alignment could exceed 1°. The authors therefore believe that narrowing the boundaries of rKA by 1°, especially where soft-tissue releases are planned, could prevent unacceptable postoperative alignment.

The technique of MA was introduced in an era when surgical instruments lacked precision.^19^ Though reproducible, it does not consider the full range of normal knee anatomy, and focuses on prosthetic survival instead.^20^ A better understanding of the functional anatomy of the knee led to the introduction of KA and rKA.^6,21,22^ Various wide and narrow coronal plane boundaries have been advocated for rKA;^2,20,23,24^ those described by MacDessi et al^15^ were used in our study. Given the potential for alterations in FCF, PTS, and FCR to affect gap balance favourably, and considering the possibility of anatomical outliers in multiplanar angular parameters, the authors used an extended version of conventional rKA, with permitted alterations in the sagittal and transverse planes.^10,25-27^ The boundaries for FCR were set between 1° of external rotation and 5° of internal rotation from the aTEA to match the described rotational boundaries of +/- 3° from the surgical transepicondylar axis (sTEA), since the sTEA lies in approximately 2° of internal rotation from the aTEA but is difficult to define intraoperatively using image-free robotics due to soft-tissue coverage.^28^

Component positioning with the NAVIO image-free robotic system and its more recent iteration, CORI, is based on the surgically planned mLDFA and mMPTA, and the resulting planned arithmetic HKAA. These are implemented by the robot, based on the femoral and tibial mechanical axes (derived using the principles of computer-assisted navigation, through the registration of bony landmarks and joint centres of the hip, knee, and ankle), and the variance of the planned angles from their respective perpendiculars. The accuracy of this system in component positioning is well-established,^29-32^ and found to be comparable or superior to image-based systems.^33,34^ Its mapping accuracy, though unclear, is relevant merely in the context of shape and size matching of planned components with the free-collection mesh during virtual surgery.

The greatest strength of our study is that the same set of knees was used for comparing the effects of E-rKA and MA, thereby eliminating the probability of errors arising from inherent anatomical differences between different sets of knees, as well as any possibility of bias. This was made possible through the use of robotic technology and virtual surgical planning. RA-TKA permitted adjustments in the sagittal and transverse planes within predefined boundaries, in addition to described strategies for coronal plane management. The primary limitation of our study also arises from the use of a single set of knees, since the effect of the decreased residual medial gap tightness could not be assessed in terms of the requirement of various soft-tissue releases, or improvements in clinical outcome scores. Another limitation of our study was that pre- and postoperative CT scans were not performed.

E-rKA is an effective and reproducible alignment strategy during TKA, enabled by robotic technology through multiplanar component adjustment possibilities. It is associated with lesser coronal gap imbalance than MA among osteoarthritic varus knees, and has the potential to manage most knees without soft-tissue releases beyond routine surgical exposure. Errors during surgical plan execution could be robot- or surgeon-related, and could occur irrespective of soft-tissue releases. Given the larger error values and the possibility of over- or under-correction of deformities during soft-tissue releases, however, alterations in component positioning within predefined boundaries would be a better starting point for correcting gap imbalance. Further research is required to assess the potential benefits of E-rKA during RA-TKA, in terms of clinical outcomes and the need for soft-tissue releases.

**Take home message**

- Extended restricted kinematic alignment is an effective and reproducible strategy during robotic-assisted total knee arthroplasty. It follows the basic principles of restricted kinematic alignment, but uses additional boundaries in the sagittal and transverse planes.

- It results in a decrease in coronal gap imbalance compared to mechanical alignment, and has the potential to manage most knees without soft-tissue releases beyond routine surgical exposure.

## Data Availability

The datasets generated and analyzed in the current study are not publicly available due to data protection regulations. Access to data is limited to the researchers who have obtained permission for data processing. Further inquiries can be made to the corresponding author.
